# Injury prevention in youth football (soccer): a comprehensive description of the development process of the ‘FUNBALL’ programme

**DOI:** 10.1136/bmjsem-2024-002260

**Published:** 2024-12-18

**Authors:** Rilind Obërtinca, Tim Meyer, Karen aus der Fünten

**Affiliations:** 1 Institute of Sports and Preventive Medicine, Saarland University, Saarbrucken, Germany; 2 Department of Physiotherapy, University of Gjakova Fehmi Agani, Gjakove, Kosovo

**Keywords:** Injury, Prevention, Football

## Abstract

Many football injury prevention programmes (IPPs) have been developed to target various injuries and populations. There was no specific IPP for youth players in place before. However, several existing IPPs designed for adults were also assessed in the youth population. All the existing ones face the challenge of long-term adherence even though efficacy has been demonstrated for many weeks to seasons. The main barriers to a successful long-term implementation of IPPs are time constraints and the lack of attractiveness for the players as they do not contain football-specific and motivating exercises. Increasing its attractiveness was the main aspect of designing the programme. To achieve this, a new approach was used. The ‘FUNBALL’ programme includes competitive, pair-based exercises and frequent ball use. It offers more flexibility as there is a choice between two different exercises for each category. It was developed through close collaboration between the research community, closely involved in real-life football, and the end-users. Tailoring IPPs to the preferences of end-users could mean a significant advancement on long-term adherence compared with previous programmes. However, further research is needed to assess this assumption.

**WHAT IS ALREADY KNOWN ON THIS TOPIC**

- Most injury prevention programmes (IPPs) in football lack sport-specific components. This is one of the main reasons for challenging long-term adherence.

**WHAT THIS STUDY ADDS**

- The ‘FUNBALL’ programme represents a new approach as it is an IPP specifically designed for youth players and employs collaboration between the research community and end-users. It integrates evidence-based practices with this population-specific needs and preferences, focusing not only on efficacy but also on applicability in real-world settings.
- The programme includes competitive exercises, frequent ball use and more exercise variation to address adherence challenges and offer flexibility for coaches and players.
- Its efficacy has been demonstrated over one season in 1027 youth football players.

**HOW THIS STUDY MIGHT AFFECT RESEARCH, PRACTICE OR POLICY**

- The new approach developing the ‘FUNBALL’ programme could increase long-term adherence, which should be investigated in future studies.

## Background

Several football injury prevention programmes (IPPs) have been developed. Some targeted region-specific injuries include groin,^1^ knee^2 3^ or thigh.^4^ Others were generic and aimed at reducing the overall number of injuries.^5^,^8^ These IPPs often focused on specific age groups. Some were designed for children^8^ and others for adults.^5^ The efficacy of the IPPs mentioned above has been reported as successful.^9^,^11^ However, based on a more detailed statistical analysis, that is, using prediction intervals in addition to the CIs and based on the quality of the evidence from existing studies, the recommendations are not unequivocal anymore.^12 13^

### Adherence challenges: football community’s resistance to IPPs

**Figure 1:**
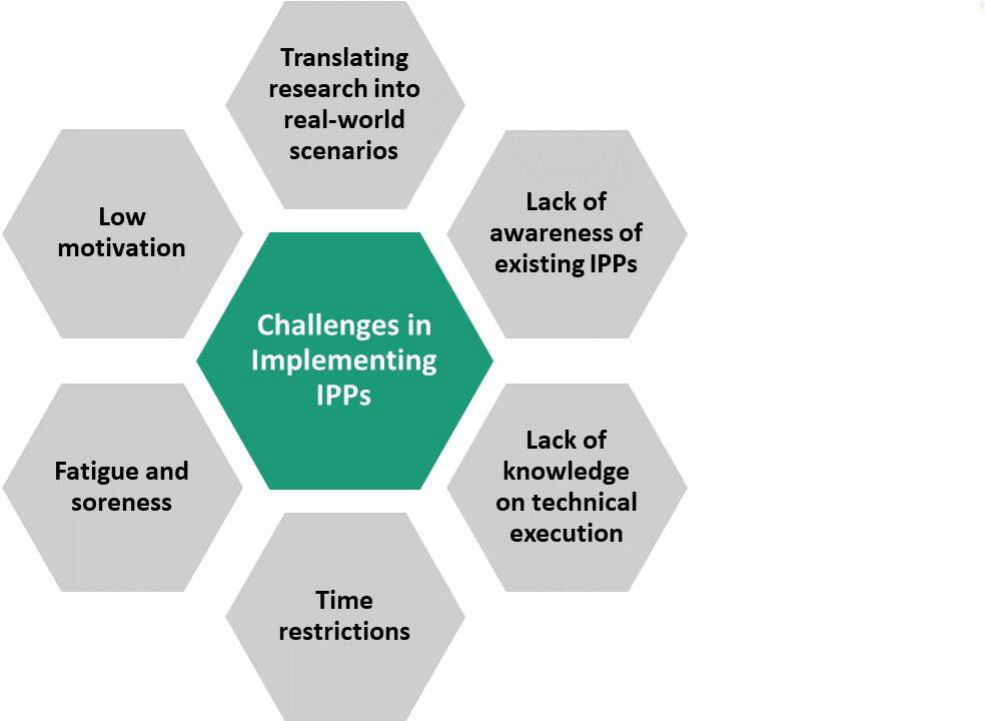
Challenges in long-term adherence for IPPs. IPPs, injury prevention programmes.

Previous research has reported several factors that influence long-term adherence to IPPs (figure 1). Translating research into real-world scenarios is a well-known challenge for all prevention programmes. Most existing IPPs have been created mainly in a research-oriented manner without considering the most suitable practical implementation. Other issues include a lack of awareness of existing programmes, insufficient knowledge on the proper technical execution of the IPP exercises and time restrictions due to other training contents.^14^,^16^ Given that most programmes are primarily designed for the use in amateur or semiprofessional football, the typical duration of their execution, usually an additional 15–20 min, is a considerable challenge in such settings. Additionally, some exercises may cause muscle fatigue and soreness, limiting their repeated use.^15 17 18^ Low motivation among players because of the absence of football-specific activities, competitiveness and exercise variability further contribute to the overall challenge of initiating and—in particular—maintaining motivation among participants.^14 19 20^

### Strategies for enhancing long-term adoption of IPPs

Despite the challenges mentioned above, a growing body of scientific research has focused on strategies to enhance compliance and adherence to IPPs over the last decade. Various aspects have been addressed, not just in football but also in other sports. Stakeholder partnership and codesigning both the IPPs and their implementation plans were established simultaneously and compliance with and adherence to them improved.^1921^,^27^ Moreover, end-users’ opinions towards IPPs and their approaches to them were investigated.^14 28^ All this work demonstrates promising progress in identifying strategies to bridge the evidence-to-practice gap.

Specifically, in football, Shamlaye et al.^14^ investigated football coaches' beliefs and attitudes about injury prevention and the 11+ programmes. A majority (57%) of coaches reported modifying the 11+ programmes to increase adherence, for example, by changing the order of the exercises or by adding a ball. Additionally, Whalan et al.^19^ explored the effect of rescheduling Part 2 of the three-part 11+ programmes on its efficacy and compliance. Their findings indicate that moving Part 2 to the end of training maintained the effectiveness of the original programme and improved player compliance. This suggests that modifications made in real-world settings may not necessarily affect the efficacy of IPPs. Finally, there is a consensus among the research community that including end-users in the development process of IPPs is important.^21^,^23^

### Rationale for the development of the ‘FUNBALL’ programme

Recent evidence suggests that (youth) football has evolved, becoming increasingly intense and physically demanding for players.^29^ However, there was no response to these changes. Neither new IPPs were developed nor existing ones were updated. What is more important is that, there was no specific IPP for youth players in place before. The negative impact of injuries in youth football is well known,^30^ and a high incidence of injuries in this age group was reported.^31^ Therefore, the intention was to develop an IPP specifically for youth players. Furthermore, the programme should contain more sport-specific components and flexibility in execution to potentially enhance long-term adherence.

### Team for development and conceptual design

**Figure 2:**
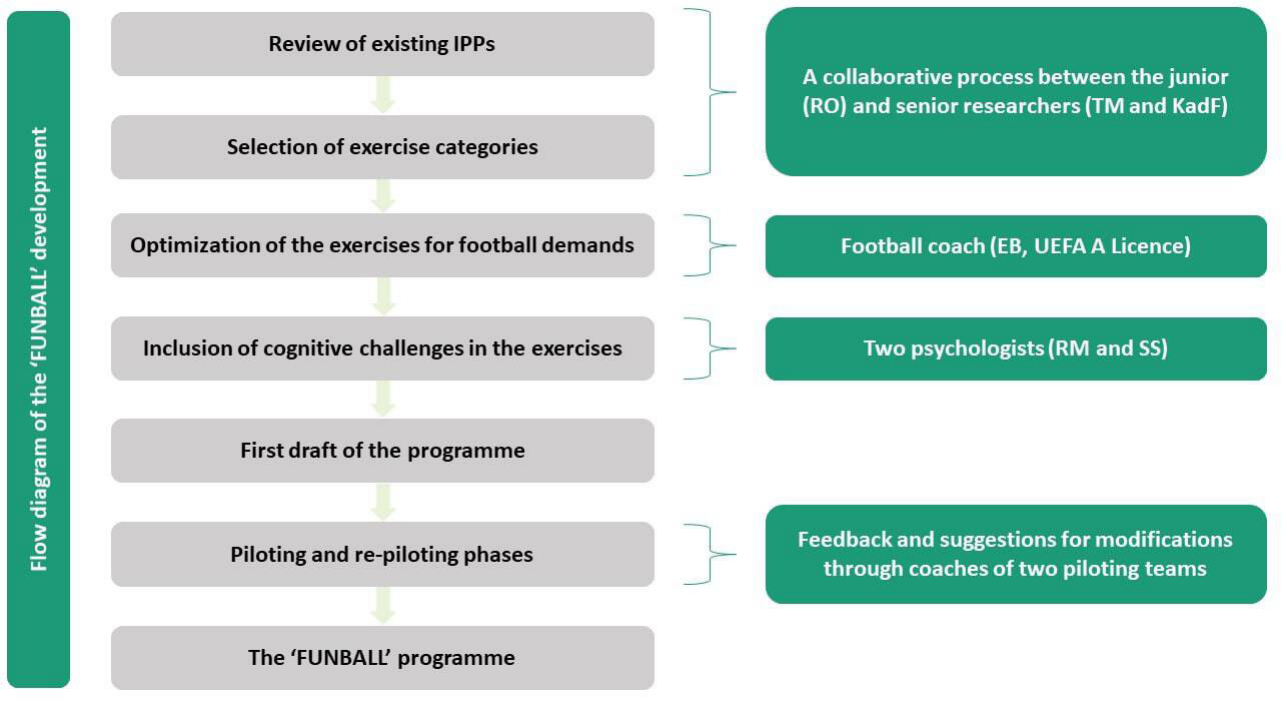
Flow diagram of the development process of the ‘FUNBALL’ programme. IPPs, injury prevention programmes.

The team that worked on the conceptual design and the development of the intervention consisted of professionals from different fields. The research team was formed as a part of RO’s PhD project. RO (sports physiotherapist; junior researcher) coordinated the project under the supervision of KadF (orthopaedic surgeon, sports medicine and chiropractor; senior researcher) and TM (sports medicine physician and sports scientist; senior researcher). Other professionals were involved in different stages of the project (figure 2): RM (neurocognitive psychologist; junior researcher) and SS (psychologist; senior researcher), finally, in the exercise planning, and EB as a football coach (UEFA A Licence).

### Journey behind the development of FUNBALL: our story

The author’s (RO) interest in football, particularly within the youth age group, stemmed from the experience working with football teams in this population. Injury prevention became a key focus due to on-field experiences with football-related injuries. Initially, the author (RO) approached the project with a practice-oriented mindset rather than a scientific one. This was mainly due to witnessing the challenges of implementing IPPs in real-world settings, particularly among youth players. Therefore, the gap between the scientific perspective and real-world implementation was evident. With this in mind and inspired by a similar idea from KadF, the author (RO) was motivated to develop a more sport-specific IPP tailored as much as possible for youth footballers. A significant advantage of this idea was that both supervisors (KadF and TM) were actively involved in football environments, particularly as team doctors, in addition to their scientific expertise. This made each step of the development project easier.

**Figure 3:**
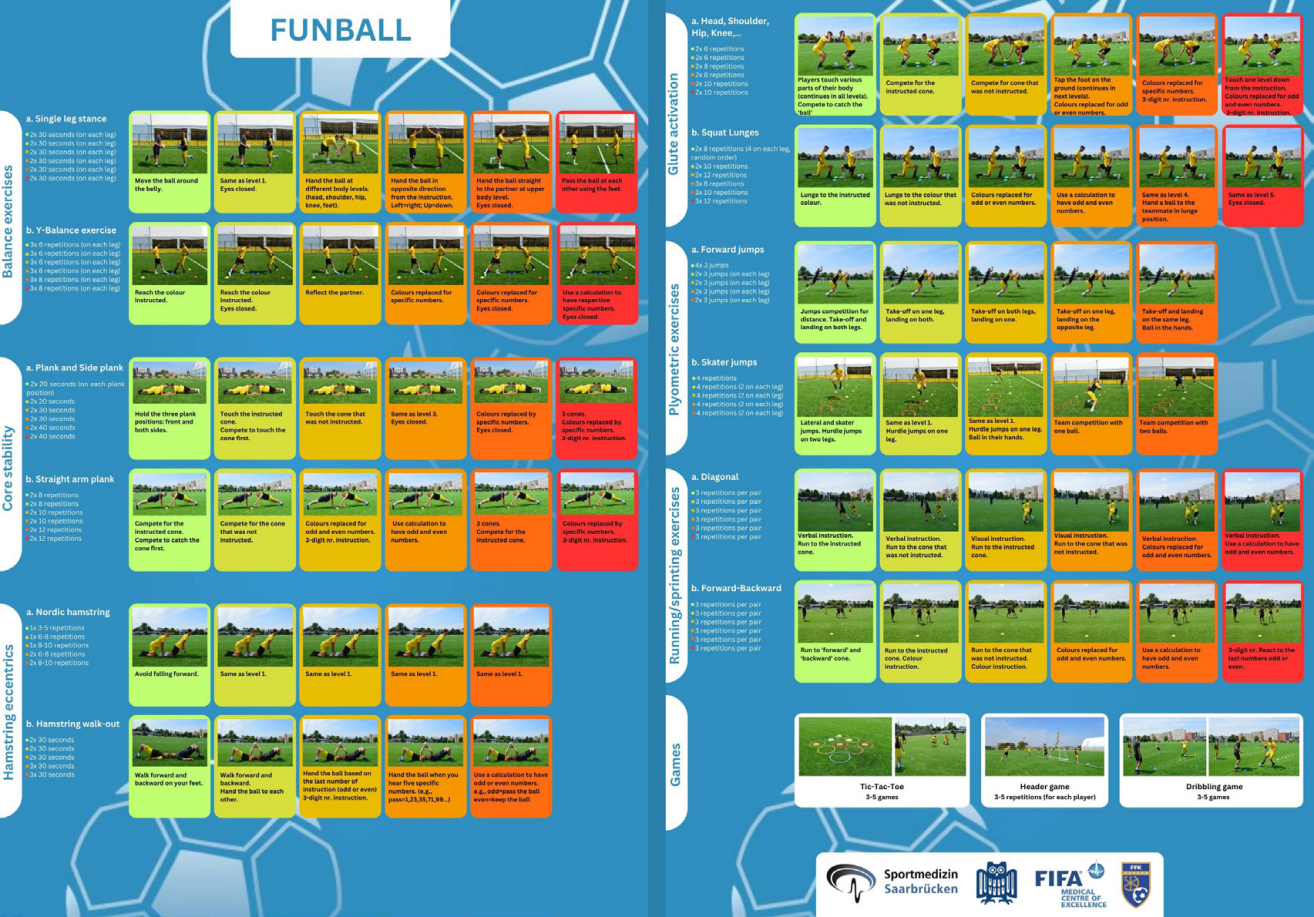
The ‘on pitch’ card of the ‘FUNBALL’ programme.

The main intention was to design more football-specific and motivating exercises, including competition between players. These exercises are performed in player pairs, with the ball included as often as possible. Moreover, the exercises contain cognitive demands, such as mental calculation, updating, inhibition and switching, reaction speed, memory and recall of information. Adding the cognitive aspect also increases the programme’s attractiveness for the players and ‘promotes multitasking’. There is also a rationale for adding a cognitive component to the exercise, as it aligns more closely with the realities and demands of the game. For example, while playing football, players simultaneously perform cognitive and physical tasks, such as running, passing the ball and observing the actions of teammates and opponents.^32^ One example: players are in pairs. They stay in a plank position facing each other. Several cones of different colours are placed between them. With instruction from the coach, they competed to touch a cone of the announced colour. The physical demands increase through the levels as repetitions and sets rise. The difficulty of the cognitive tasks also increases. This can include adding inhibition or mental calculation tasks, such as the coach replacing the cone colour with odd or even numbers and then using calculations to identify odd and even numbers. A detailed description of all the exercises included is presented in figure 3.

Additionally, we aimed to provide more flexibility for coaches and players, allowing them to choose between two exercises for each exercise category. Finally, unlike the existing IPPs, the idea was to provide a programme that coaches could use after their usual warm-ups, as coaches often prefer established warm-ups, which they do not like to change. Such a change could, otherwise, have reduced compliance and all protagonists' (long-term) adherence. However, we aimed to follow the existing practice of IPPs by designing exercises with progressive difficulty levels.

The development of the programme required a collaborative effort (figure 2). Initially, the Football Federation of Kosovo was involved. When the idea to develop the programme was presented to them, we received positive feedback regarding their support with logistics, the distribution of the programme and the coordination of its implementation across the Kosovo Youth Leagues for the U15–U19 teams.

We aimed to integrate best evidence practices while at the same time enhancing sport specificity. The programme’s design was grounded in the theoretical framework of translating research into injury prevention practice.^33^ The starting point was a review and meta-analysis of the existing multicomponent, exercise-based IPPs.^12^ The exercise categories for the new programme were chosen based on this detailed review. Additionally, recognising the importance of sport specificity and following recommendations from previous research on adherence challenges,^21 24^ we brought a football coach onto the development team. His role was crucial in optimising the selected exercises to better align with the demands of football. Later, during the planning phase, two psychologists were included. Their task was to increase the cognitive challenge of the chosen exercises.

The initial selection of ideas was a collaborative process between the lead investigator and senior researchers. We then worked closely with the football coach to refine these exercises. This phase involved significant revisions. Several exercises were modified to increase their competitiveness or even removed from the programme.

Following the development phase, the programme was piloted with a football team in the under-15 age group. This piloting was initially done 'per exercise category', meaning we designed one exercise category, such as 'balance exercises', and then tested it with the team. The same process was followed for the other exercise categories. After each training session, we held meetings with the coaches of the piloting teams to obtain feedback. Some exercises were found to be too lengthy, and feedback indicated a need for adjustments to enhance their acceptance. Additionally, a few exercises were considered too difficult for youth players, especially for those in the under-15 age group. Subsequently, the coach involved in the developing programme suggested modifications or replacements for those exercises, which were then presented to the piloting team coaches. The exercises were modified and/or replaced until all coaches approved the changes.

Afterwards, the first draft of the entire programme was completed. We chose another team in the under-17 age group for the second pilot phase in which players had to perform all exercise categories. The coaches of this age group suggested changing one exercise, as it was reportedly too time-consuming. Following the above-mentioned process, we changed and repiloted the entire programme several times with the same team. After a few implementation rounds of the entire programme with the under-17 team, we received positive feedback from the coaches and decided to close the programme’s development phase. Unfortunately, we did not include any players in the 'team that developed' the programme. However, their feedback during the piloting phases was collected through coaches.

### Efficacy testing

The programme’s efficacy in injury reduction was investigated through a large cluster randomised controlled trial (cluster-RCT), which included 1027 players (aged 13–19 years) from semiprofessional youth leagues in Kosovo. When applied over an entire football season, the programme reduced the incidence of overall football-related injuries, thigh injuries and moderate to severe injuries. A detailed description of the efficacy of 'FUNBALL' and access to the full version of the programme is available elsewhere.^34^ Moreover, positive effects on the cognitive performance of the players in the intervention group of the aforementioned cluster-RCT were reported.^25^ Several cognitive abilities were improved, including working memory, visual learning, visual motor control, attention, psychomotor function, memory and executive function.

## Conclusion

In summary, the development of the ‘FUNBALL’ programme represents a step forward in tailoring IPPs to meet the sport-specific demands of footballers. Its efficacy has been reported as successful; however, whether including sport-specific components will positively affect long-term adherence warrants further investigation.
